# The Differences in Expression of Tumor Marker KI-67 and Proliferation Cell Nuclear Antigen (PCNA) in Ameloblastoma with Histopathological Subtypes Follicular, Plexiform, and Mixed (Follicular-Plexiform)

**DOI:** 10.1055/s-0044-1791220

**Published:** 2025-05-15

**Authors:** I. Gusti Putra Swabuana Purwoyudho, David Buntoro Kamadjaja, R.M. Coen Pramono Danudiningrat, Muhammad Subhan Amir, Andra Rizqiawan, Indra Mulyawan, Okky Prasetio

**Affiliations:** 1 Department of Oral and Maxillofacial Surgery, Faculty of Dental Medicine, Universitas Airlangga, Surabaya, Indonesia; 2 Faculty of Medicine, Universitas Airlangga, Surabaya, Indonesia; 3 Department of Oral and Maxillofacial Surgery, Faculty of Dental Medicine, Academic Dental Hospital, Universitas Airlangga, Surabaya, Indonesia; 4 Department of Oral and Maxillofacial Surgery, Universitas Airlangga Hospital, Surabaya, Indonesia; 5 Department of Oral and Maxillofacial Surgery, Dr. Mohamad Soewandhie Regional General Hospital, Surabaya, Indonesia

**Keywords:** ameloblastoma, follicular, plexiform, Ki-67, PCNA, immunohistochemistry

## Abstract

**Objective:**

Ameloblastoma, though classified as a benign tumor, can induce deformities and functional abnormalities in the craniofacial region. Ki-67 is a typical marker for cell proliferation, with its expression peaking the M phase of the cell cycle or mitosis, indicating active cell division. Cells with high levels of Ki-67 expression are typically in a state of active proliferation. In addition, proliferating cell nuclear antigen (PCNA), a nuclear protein, plays a role in regulating the cell cycle and is involved in deoxyribonucleic acid (DNA) replication and repair processes. PCNA acts as a cofactor in DNA replication and repair processes.

**Material and Methods:**

This study used 37 postoperative paraffin blocks for ameloblastoma patients in the period of 2015 to 2023. The samples in the inclusion criteria involved 24 samples with ameloblastomas diagnosis. Immunohistochemistries were used to observe the expression of Ki-67 and PCNA.

**Results:**

The lowest expression of Ki-67 and PCNA was found in the plexiform subtype, whereas the highest expression values were found in mixed subtype (follicular-plexiform). The Tukey honestly significant difference test indicated a significant difference in Ki-67 expression in mixed subtypes compared to the plexiform and follicular types, with values of 0.001 ( *p* < 0.05), and for PCNA expression was found with significant difference, 0.001 and 0.000 ( *p* < 0.05), in the mixed subtypes, higher compared to the follicular and plexiform type.

**Conclusion:**

There is a significant difference in the expression values of Ki-67 and PCNA in the follicular subtype when compared to the plexiform subtype. However, no significant difference in values was observed between the follicular type and the mixed type.

## Introduction

**Fig. 1:**
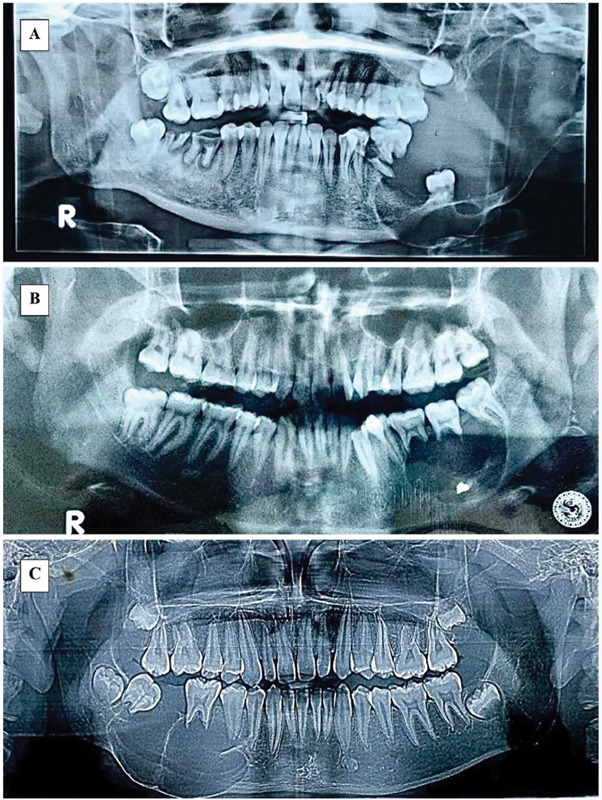
Radiograph examination of ameloblastoma. ( **A** ) A 22 years old female patient with histopathological diagnosis of follicular ameloblastoma. ( **B** ) A 22 years old male patient with histopathological diagnosis of plexiform ameloblastoma. ( **C** ) A 14 years old male patient with histopathological diagnosis of follicular-plexiform ameloblastoma.

Ameloblastoma is a benign odontogenic tumor that grows in the jawbone, slowly but is locally aggressive with clinical manifestations in the form of enlargement of the jaw area. It often does not cause complaints of pain, and can expand to the cortical bone. 1 Many theories have emerged regarding the etiology and growth pattern of ameloblastoma. Neville et al stated that the possible pathogenesis of ameloblastoma growth arises from (1) the remaining cells of the enamel organ, either from the remaining dental lamina or sheath of Hertwig, (2) the developing enamel organ, (3) basal cells from the forming jaw epithelium, (4) heterotrophic epithelium from other parts of the body, especially the pituitary gland, (5) epithelium from cysts, especially dentigerous cysts, (6) rest of Serres epithelial cells in the gingiva, and (7) basal cells from the oral mucosa, the result of basal cell invagination of epithelium to the developing jawbone. 2 One of the basic supportive examinations required to establish the diagnosis of ameloblastoma is radiography ( Fig. 1 ).

Ameloblastoma is classified according to clinical features, radiology, therapeutic considerations, and histopathology. Based on the World Health Organization (2017) classification regarding ameloblastoma histological subtypes, ameloblastoma is divided into six subtypes, including (1) follicular, (2) plexiform, (3) acanthomatous, (4) basal cell ameloblastoma, (5) granular cell ameloblastoma, and (6) desmoplastic types. 3 The ameloblastoma patterns often found in the microscopic picture are follicular and plexiform patterns. 3

The underlying mechanisms of ameloblastoma are still a subject of debate. Cellular changes are influenced by several factors involved in tumor growth and tumor biology. Several studies have divided the function of molecular markers into eight types: (1) markers involved in extracellular matrix (ECM) degradation (matrix metalloproteinase [MMP]-2, MMP-14, tissue inhibitor of metalloproteinases 2), (2) loss of cell adhesion and involvement in cell migration (CD-138/Ki-67, proliferating cell nuclear antigen [PCNA]), (3) bone resorption and remodeling (PTHrP, RANKL, OPG), (4) cell proliferation in ameloblastoma (P16, Ki-67, PCNA), (5) function of stromal tumor cells in ameloblastoma invasion (CD-133, Bmi-1), (6) tumor growth and angiogenesis (fibroblast growth factor, vascular endothelial growth factor [VEGF]), (7) apoptotic function (Fas ligand, caspase-3, survivin), and (8) tumor suppression (P53, phosphatase and tensin homolog). 4

Research in immunostaining of various lesions of odontogenic origin has improved our understanding of the developing tooth germ immunophenotype, as well as the application of tumor markers that can provide a more in-depth picture of epithelial or mesenchymal tumors. Ki-67 protein has been extensively investigated as a potential prognostic marker of proliferation in retrospective studies of malignant diseases. Accumulating clinical studies have demonstrated Ki-67 as a tool for cancer diagnosis. Ki-67 is useful in determining the proliferation index of cells, which is the percentage of cells in a tissue sample that are positive for Ki-67. 5 A high Ki-67 index indicates a high level of cell proliferation and is often associated with aggressive tumor behavior. 6 In various cancers, including ameloblastoma, a higher Ki-67 labeling index is generally associated with more aggressive disease, increased likelihood of metastasis, and poorer prognosis. It helps in assessing how fast the tumor is growing. 7 High Ki-67 expression in ameloblastoma may correlate with a higher likelihood of local recurrence and may inform treatment strategies. 8

Meanwhile, PCNA is also a nuclear nonhistone protein required for deoxyribonucleic acid (DNA) synthesis and is an additional protein for DNA polymerase alpha, which increases during the G1/S phase of the cell cycle. 9 PCNA expression can be used as a marker of cell proliferation because cells remain in the G1/S phase longer when proliferating. Furthermore, this protein has an important role in nucleic acid metabolism as a component of DNA replication and repair mechanisms. 10 Such Ki-67, PCNA is used to identify cells that are actively synthesizing DNA, thus providing a measure of the proliferative activity of a tissue. It helps in understanding how many cells are in the DNA synthesis phase, which can be indicative of overall cell proliferation. 11 PCNA expression in ameloblastoma helps evaluate the proliferative activity of the tumor. High levels of PCNA can indicate more aggressive tumor behavior, higher recurrence rates, and potentially poorer prognosis. 12

Geographical and demographic factors are able to influence histopathological features, including markers of Ki-67 and PCNA. These markers can be affected by various factors related to geography and patient characteristics. Studies have indicated that variations in Ki-67 and PCNA expression can be associated with different populations and geographical regions, reflecting underlying genetic and environmental factors. Different populations may have genetic variations that affect the expression of Ki-67 and PCNA. For instance, genetic predispositions to certain types of cancer or variations in cell cycle regulation genes can influence these markers. Exposure to environmental factors, such as pollution, diet, and lifestyle, can impact cancer development and progression. These factors can influence cell proliferation rates and, consequently, the expression of Ki-67 and PCNA. 13

## Materials and Methods

### Study Design and Population

The data were collected from ameloblastoma paraffin blocks sourced from the anatomy and pathology department of Dr. Mohamad Soewandhie Regional General Hospital and Airlangga University Hospital between 2015 and 2023. This research utilized total sampling technique because the population was less than 100 participants. The study was ethically approved, adhering to the Declaration of Helsinki principles.

### Data Collection

Data collection was tabulated from medical records, including age, gender, and postoperative histopathology.

Inclusion criteria:

1. Paraffin blocks from postoperative ameloblastoma patients from Dr. Mohammad Soewandhie and Airlangga University Hospital, 2015 to 2023 as the representative.
2. Postoperative biopsy result was noncarcinoma ameloblastoma, enough tissue for examination, and there was a completed medical record.
3. Paraffin blocks from postoperative ameloblastoma patients and the histopathological subtypes were follicular, plexiform, and mixed type.

Exclusion criteria:

1. Paraffin block of postoperative ameloblastoma patients and histopathological subtypes other than follicular, plexiform, and mixed subtypes (follicular and plexiform) were obtained.
2. Blocks of postoperative ameloblastoma patients which did not represent the ameloblastoma picture in the form of tumor parenchyma and stroma so that immunohistochemical (IHC) examination cannot be carried out.

### Immunohistochemical Staining

Ki-67 antibody (Ki-67: sc-23900 Concentrated and Prediluted Mouse Monoclonal, Human Reactivity, Santa Cruz Biotechnology, Europe) and PCNA antibody (PC-10: sc-56 Concentrated and Prediluted Mouse Monoclonal, Human Reactivity, Santa Cruz Biotechnology, Europe) were used. Counterstaining was carried out using Mayer Hematoxilen and incubated for 20 minutes. Washed with running water, dipped in aquadest, air dried at room temperature, implanted using Entellan, and covered with a cover glass. Slides that had been stained with IHC antibodies were observed with an Olympus BX53 light microscope. The slides were covered with the code and provided a new number randomly. With 400× and 1000× magnification, the tumor epithelial cells were counted, which were brown in the cytoplasm for Ki-67 and in the cell nucleus for PCNA. The amount of Ki-67 and PCNA expression was calculated from the average of 20 visual fields and presented as the mean ± standard deviation. Visual fields were documented with a Sony ZV10 camera.

### Statistical Analysis

Descriptive data was presented to determine the distribution of the data obtained. Data presentation was in the form of frequency distribution tables and bar or pie charts. Tables and diagrams show the distribution of gender, age, and ameloblastoma histopathological type variants. Data analysis began with a normality test of the Ki-67 and PCNA expression measurement results for each group using the Shapiro–Wilk test. The next comparison test used the parametric one-way analysis of variance test to determine differences in Ki-67 and PCNA expression in the three groups. Further tests ( *post hoc* ) carried out multiple comparison analyses of Tukey honestly significant difference to determine differences in Ki-67 and PCNA expression between groups.

## Results

**Fig. 2:**
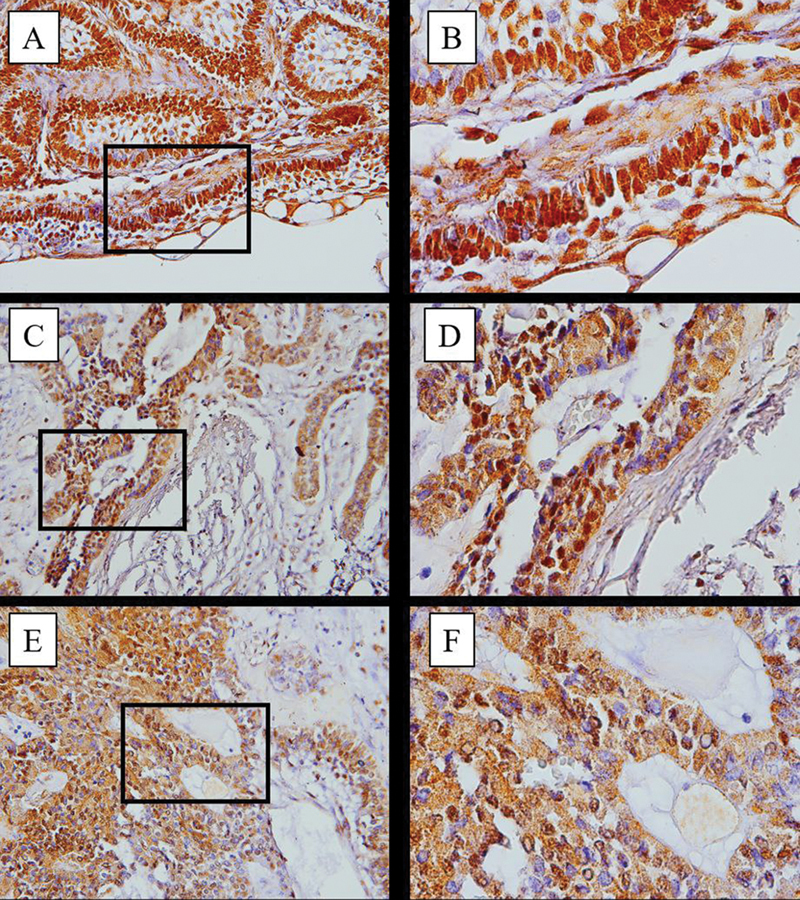
Results of anti-Ki-67 immunohistochemical staining on ameloblastoma samples. ( **A** ) A 22 years old female patient with follicular type 400× magnification. ( **B** ) A 22 years old female patient with follicular type 1000× magnification. ( **C** ) A 22 years old male patient with plexiform type 400× magnification. ( **D** ) A 22 years old male patient with plexiform type 1000× magnification. ( **E** ) A 14 years old male patient with follicular-plexiform type 400× magnification. ( **F** ) A 14 years old male patient with follicular-plexiform type 1000× magnification.

Based on the image of IHC staining results on samples (magnification 400× – 1000×), brown stained cells indicate that express Ki-67 ( Fig. 2 ). This expression located in tumor epithelial cells from each ameloblastoma sample. In the follicular and mixed subtypes (follicular-plexiform), Ki-67 appears to be more expressed than in the plexiform subtype. The measurement of Ki-67 expression using the IHC test showed that the average Ki-67 expression value was highest in the mixed subtype group (follicular-plexiform) among the ameloblastoma subtypes in this study sample. Specifically, the mean value of Ki-67 expression values, ranked from highest to lowest, were as follows: the mixed type (follicular-plexiform) at 45.22, the follicular subtype at (40), and the plexiform subtype showing the lowest expression at 31.08 ( Table 1 ).

**Table 1:** Distribution of Ki-67 and PCNA expression and multiple comparison *post* *hoc* Tukey HSD in ameloblastoma subtypes

Marker	Subtype	*N*	Mean	Standard deviation	Compared to	*p* -Value
Ki-67	Follicular	4	40	5.2915	Plexiform	0.001
					Follicular-Plexiform	0.076
	Plexiform	12	31.0833	3.11764	Follicular	0.001
					Follicular-Plexiform	0.000
	Follicular-Plexiform	8	45.2222	3.89801	Follicular	0.076
					Plexiform	0.000
PCNA	Follicular	4	31	5.65685	Plexiform	0.001
					Follicular-Plexiform	0.439
	Plexiform	12	22.25	3.04884	Follicular	0.001
					Follicular- Plexiform	0.000
	Follicular-Plexiform	8	33.7778	3.59784	Follicular	0.439
					Plexiform	0.000

Abbreviations: HSD, honestly significant difference; PCNA, proliferating cell nuclear antigen.

**Fig. 3:**
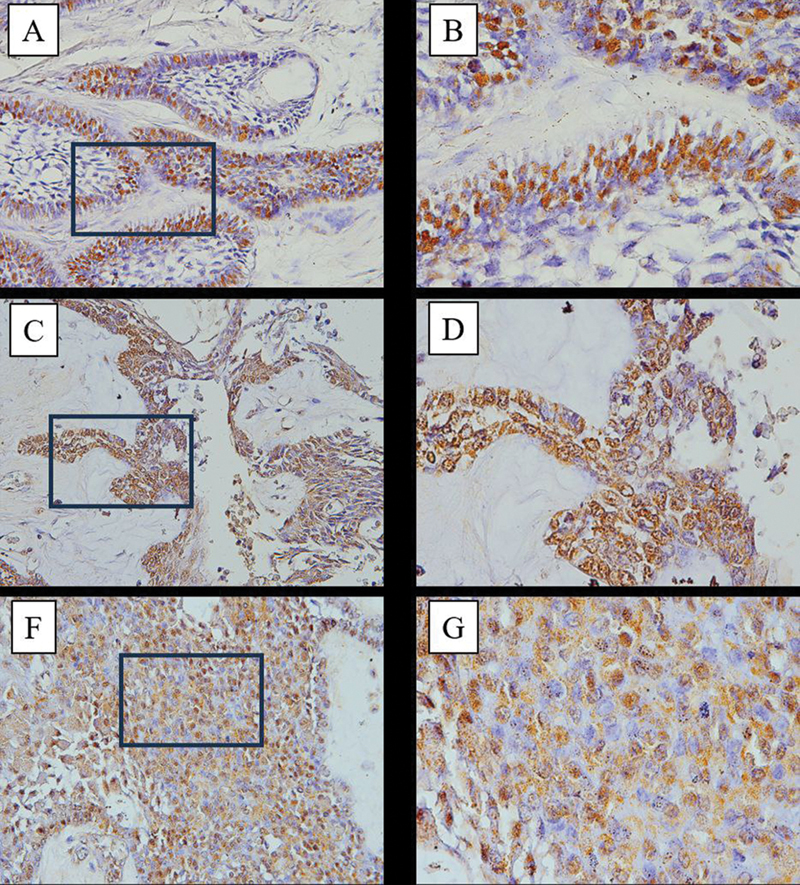
Results of proliferating cell nuclear antigen (PCNA) immunohistochemical staining on ameloblastoma samples. ( **A** ) A 22 years old female patient with follicular type 400× magnification. ( **B** ) A 22 years old female patient with follicular type 1000× magnification. ( **C** ) A 22 years old female patient with plexiform type 400× magnification. ( **D** ) A 22 years old female patient with plexiform type 1000× magnification. ( **E** ) A 14 years old male patient with follicular-plexiform type 400× magnification. ( **F** ) A 14 years old male patient with follicular-plexiform type 1000× magnification.

Based on the image of IHC staining results on samples (magnification 400× – 1000×), brown stained cells indicate that express PCNA ( Fig. 3 ). PCNA expression was located in tumor epithelial cells from each ameloblastoma sample. PCNA expression was observed to be lower in the plexiform subtype than in the follicular and mixed subtypes (follicular-plexiform). The average PCNA expression values, ranked from highest to lowest value, were mixed subtype (follicular-plexiform) at 33.77, follicular subtype at 31, and plexiform subtype at 22.25. Similarly, the mean value of Ki-67 expression value followed a descending order with the mixed type subtype (follicular-plexiform) at 45.22, follicular subtype at 40, and the lowest is in the plexiform subtype at 31.08 ( Table 1 ).

The results of the Ki-67 comparison test between the follicular and plexiform groups showed a significance value ( *p* ) of 0.001, which means there is a significant difference between the ameloblastoma subtype groups. In the comparison test between the follicular and mixed (follicular-plexiform) subtype groups, the significance value ( *p* ) was 0.076, which means there was no significant difference between the groups. In the PCNA comparison test between the follicular and plexiform subtype groups, a significance value ( *p* ) of 0.001 was obtained, which means there was a significant difference between the groups. Meanwhile, in the comparison test between the follicular and mixed subtype groups (follicular-plexiform), the significance value ( *p* ) was 0.439, meaning that there was no significant difference between the groups ( Table 1 ).

## Discussion

Ameloblastoma is a benign tumor that can invade the surrounding local area. Cellular invasion of the tumor requires damage to the basement membrane and ECM around the tumor, followed by the growth and proliferation of tumor cells. Decreased adhesion between cells and the ECM and changes in basement membrane composition are also associated with the growth of malignant neoplasms. In the neoplastic process, abnormal and uncontrolled cell proliferation, as well as changes in the cell cycle, becomes the important phenomena to observe. Assessment of cell proliferation activity in tumors has become a common tool used by histopathologists to provide useful information for assessing and predicting tumor behavior, namely, the likelihood of local recurrence, its metastatic potential, and the growth of metastases, as well as the prognosis of how long the survival rate is (probability of survival), which is free of tumor recurrence, as well as the survival rate period until death. 14

The increased Ki-67 expression in ameloblastoma is also correlated to hypoxia in the tumor, where the use of large amounts of energy and oxygen by tumor cells results in intratumoral hypoxia. This condition triggers the release of hypoxia-inducible factor 1-alpha (HIF-1α) as a form of adaptation of cells in hypoxic conditions. 15 The role of HIF-1α is also as an agent that directs the migration of mature endothelial cells toward hypoxic environments by regulating VEGF transcription. Hypoxic inducible factor 1 bind to the VEGF gene, inducing its transcription and expression. 16 HIF-1α expression is slightly higher in follicular ameloblastoma than in plexiform. Therefore, it may contribute to VEGF expression differences. 17 In the process, angiogenesis is influenced by the degradation of the ECM, which is triggered by MMP activity and proteolytic enzymes, which cause the release of angiogenic growth factors from the basement membrane of blood vessels. 18 Research conducted by Setiawan et al indicates that the expression of MMP-2, MMP-9, and interleukin-1α is the highest in the follicular type, followed by plexiform and mixed. 19

Some studies suggest that PCNA expression levels may be increased in more aggressive ameloblastoma cases. This increased PCNA expression may indicate higher cell proliferation activity in the tumor. High cell proliferation may contribute to more aggressive tumor growth. High PCNA expression may be associated with a worse prognosis or a higher risk of recurrence. 20 Research by Hertog et al showed that the highest recurrence rate in ameloblastoma undergoing conservative therapy was found in the follicular subtype (7/10), followed by the mixed subtype (3/7) and the plexiform subtype (4/11). This may also be related to increased expression of Ki-67 and PCNA, where the follicular subtype has the appearance of cells arranged in a palisading manner and reverse nuclear cell polarity appears. 21

The level of Ki-67 and PCNA expression can help pathologists differentiate between subtypes of ameloblastoma and assess their potential behavior. This can be crucial for accurate diagnosis and anticipate how the tumor might progress. 22 Moreover, elevated Ki-67 and PCNA levels can help distinguish ameloblastoma from other odontogenic tumors or lesions with similar histological features but different clinical behaviors. 23 Higher Ki-67 and PCNA levels are associated with a higher risk of local recurrence. This information can be used to guide patient counseling and plan more intensive follow-up strategies. The proliferation markers can also provide insights into how well a tumor is responding to treatment, particularly if nonsurgical therapies, which are being considered or if adjuvant therapies are required, understanding these variations helps in tailoring personalized treatment plans and improving patient outcomes. 24

Due to its retrospective nature, this study still has certain limitations, including the limited sample size and the absence of data on patient samples' follow-up in the form of tumor-free survival time and recurrence data.

## Conclusion

The results of the research data analysis revealed that the highest expression of KI-67 and PCNA was found in the follicular and mixed ameloblastoma subtypes (follicular-plexiform) compared to the plexiform subtype. There are significant differences in the expression of KI-67 and PCNA in the follicular subtype and mixed subtype (follicular-plexiform) with the plexiform subtype (follicular-plexiform). As a result, with a limited number of samples, this shows that the expression of Ki-67 and PCNA in the follicular and mixed type ameloblastoma (follicular-plexiform) is higher than in plexiform subtype ameloblastoma.
